# T cell exhaustion in pediatric B-ALL: current knowledge and future perspectives

**DOI:** 10.3389/fimmu.2025.1531145

**Published:** 2025-05-28

**Authors:** Tanmaya Atre, Gregor S. D. Reid

**Affiliations:** ^1^ Michael Cuccione Childhood Cancer Research Program, BC Children’s Hospital Research Institute, Vancouver, BC, Canada; ^2^ Department of Pediatrics, University of British Columbia, Vancouver, BC, Canada

**Keywords:** pediatric B cell precursor acute lymphoblastic leukemia, T cell exhaustion (Tex), bispecific T cell engager (BiTE), chimeric antigen receptor (CAR T), immune checkpoint inhibition

## Abstract

B-cell acute lymphoblastic leukemia (B-ALL) is the most common pediatric malignancy, accounting for 20-25% of all new cancer diagnoses in North American children each year. The leukemia arises, most commonly after a latency of 3–5 years, from a preleukemic B cell precursor population generated *in utero*. Despite the generally low immunogenicity of B-ALL cells, emerging evidence implicates T cell exhaustion - a state marked by sustained expression of inhibitory receptors and progressive functional decline - as a contributor to disease progression. Expression of inhibitory receptors is frequently detected on T cells from children with B-ALL at diagnosis and during therapy. As T cell exhaustion presents an actionable target for enhancing protective immune activity, in this review we summarize evidence from both clinical and pre-clinical settings for T cell exhaustion during pediatric B-ALL progression and discuss the opportunities and challenges to incorporating immune checkpoint blockade into pediatric B-ALL therapy regimens.

## 1 Introduction

T cell activation is initiated when T cell receptor (TCR) engagement with peptide-MHC complexes on antigen-presenting cells (APC) (signal 1) is accompanied by co-stimulatory signals (signal 2) and cytokine support (signal 3). This coordinated signaling cascade drives lymphocyte activation, proliferation, and differentiation that mediates antigen clearance (1, 2). Typically, immune checkpoints (IC) then regulate the immune response by engaging with inhibitory receptors on activated T cells (3, 4). The timing and balance of stimulatory and inhibitory signals influences the quality and duration of T cell responses (5). However, in cases where antigen clearance is not achieved, prolonged T cell stimulation can lead to an altered differentiation state, known as ‘*exhaustion*’ (6). T cell exhaustion is characterized by sustained upregulation and co-expression of multiple inhibitory IC receptors, along with transcriptional (7), metabolic (8), and epigenetic modifications (9), resulting in a progressive loss of effector function in antigen-specific T cells (10). The accumulation of exhausted T cells is observed in many cancers (11).

The genomic alterations that drive tumor formation can lead to the generation of immunogenic neoantigens (12). Through iterations of the tumor-immune cycle, recognition of these altered-self epitopes by the adaptive immune system can initiate and maintain immune responses that exert ongoing immunosurveillance (13, 14). However, if these responses fail to eliminate the nascent tumor, exposure to an increasing burden of neoantigens can lead to T cell exhaustion and downregulation of protective immune activity, leading to tumor progression (15, 16). The development of immune checkpoint blockade (ICB) therapy to overcome inhibitory signaling pathways and re-establish T cell-mediated anti-tumor activity has markedly transformed the therapeutic landscape for several human malignancies (17–19). While outcomes achieved with ICB therapy have been unprecedented, there is a growing recognition from adult cancer studies that properties of the patient’s immune system can significantly influence the outcome of these therapies (20, 21). Whether these same immune variables will modulate responses to ICB in pediatric cancer patients remains largely unknown.

**Figure 1:**
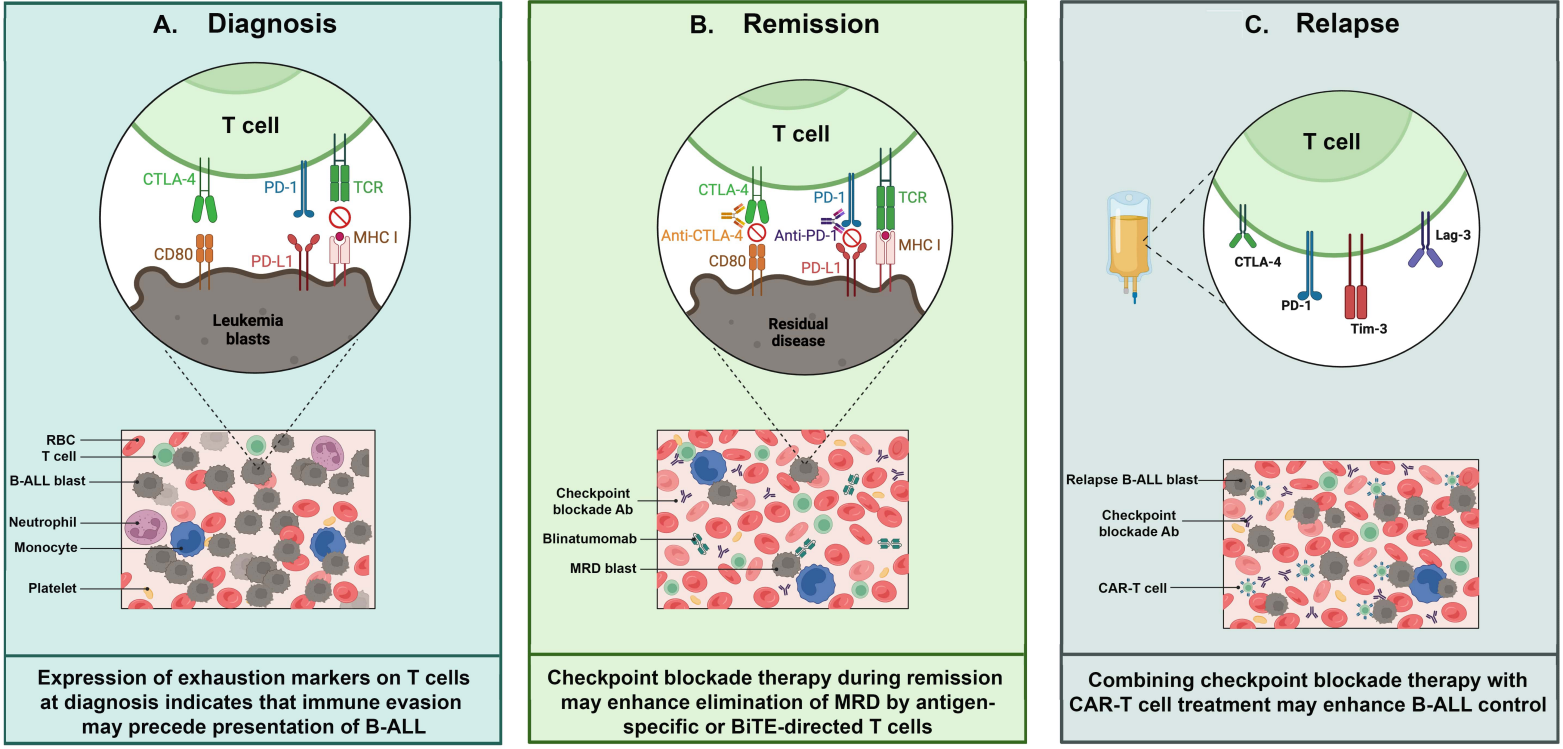
T cell exhaustion in pediatric B-cell acute lymphoblastic leukemia (B-ALL). Exhausted T cells can be detected and targeted at various timepoints during B-ALL progression: **(A)** Diagnosis: Exhausted T cells in bone marrow aspirates from patients at diagnosis express multiple inhibitory receptors such as PD-1,TIM-3 and CTLA-4, which impair T cell function. These receptors interact with their respective ligands expressed on leukemia blasts, contributing to immune evasion. **(B)** Remission: When leukemia burden is reduced to minimal residual disease (MRD) levels by induction chemoptherapy, there is an opportunity to restore anti-leukemia immunity. At this stage, immune checkpoint inhibitors - e.g. anti-PD-1 and anti-CTLA-4 antibodies (Abs) - may reinvigorate T cells, especially when combined with approved immunotherapies such as CD3/CD19 bispecific T cell engagers. **(C)** Relapse: CAR-T cells show an exhausted phenotype, characterized by high expression of inhibitory receptors including PD-1, TIM-3, LAG-3, and CTLA-4, at various times during production and application. ICB may enhance CAR-T functionality, but the optimal timing to achieve this remains to be determined. Abbreviations – Minimal residual disease (MRD); Bispecific T cell engager (BiTE); Chimeric antigen receptor (CAR); red blood cell (RBC); antibody (Ab).

Pediatric cancers generally exhibit a lower tumor mutation burden (TMB) than adult cancers (22–24), likely resulting in reduced neoantigen-driven immune stimulation and infiltration. Notably, ICB treatment responses are strongest for those rare pediatric cancers that possess TMB approaching or exceeding those of adult tumors (25, 26). Furthermore, many pediatric malignancies, such as B cell acute lymphoblastic leukemia (B-ALL), neuroblastoma, Wilm’s tumor, and medulloblastoma, have a prenatal origin and manifest in early childhood following a relatively short latency period (27). Fetal and neonatal development are times of extensive immune tolerance induction (28, 29), suggesting that the early genetic alterations driving pediatric cancers may evade immune detection. Given these potential constraints on the effectiveness of checkpoint inhibition in the setting of childhood cancer, this review examines the evidence supporting the application of ICB therapy to improve outcomes for children with B-ALL, the most common of the prenatally initiated pediatric cancers (**Figure 1**).

## 2 B-ALL immunogenicity

B-ALL comprises a heterogeneous group of malignancies characterized by the uncontrolled proliferation of B cell progenitors within the bone marrow (30). It is the most common pediatric malignancy, accounting for almost a quarter of childhood cancer cases worldwide (31, 32). While the incidence of B-ALL peaks between 3 and 5 years of age, the initiating event usually occurs *in utero*: more than 70% of patients have detectable leukemia-initiating cytogenetic abnormalities at birth (33–37). Full transformation to overt leukemia requires additional genetic lesions to occur within the early arising preleukemic population (38). Treatment advancements have significantly improved outcomes for children with B-ALL, with the current 5-year event-free survival rate exceeding 85% in North America (39, 40). Nevertheless, around 20% of patients will remain unresponsive to initial therapy or experience relapse (41, 42). For these patients, targeted immune therapies, such as chimeric antigen receptor (CAR) T cells and bi-specific T cell engagers (BiTE), offer considerable hope (43, 44). However, achieving more durable responses with these novel treatments has emerged as a clinical imperative (45, 46).

The mutation burden in pediatric B-ALL is relatively low, ranging from 0.15 to 0.66 single nucleotide variants (SNVs) per megabase (22), a frequency predicted to result in relatively few neoantigens (47, 48). Additionally, B-ALL blasts often lack or have limited expression of essential co-stimulatory molecules, including CD80 and CD86 (49, 50). The combination of weak neoantigen expression and poor co-stimulation is predicted to favor induction of T cell anergy over activation. Consistent with this prediction, early studies reported that antigen presentation by B-ALL blasts often induces T cell deletion or anergy (51–54). The low immunogenicity of B-ALL, however, is not absolute, as ALL-specific CD4+ and CD8+ T cell responses can be generated under experimental conditions, indicating that the leukmeic blasts can present immunogenic epitopes (55, 56). Subsequent research demonstrated that 88% of B-ALL cases contain at least one predicted neoepitope (48). Further, leukemia antigen-specific T cells can be generated from pediatric B-ALL patients during maintenance therapy, and these T cells exhibited cytotoxicity against autologous leukemia cells (57). High-throughput studies further support this finding, showing that CD8+ T cells from patients with B-ALL can recognize and respond to neoantigens derived from fusion proteins, such as ETV6::RUNX1 (58)

Mouse models of B-ALL further support the feasibility of T cell-mediated control over leukemia, although these studies rely primarily on leukemia transplant approaches that do not capture the potential impact of neonatal tolerance. In a recent study using a murine *Arf* ^−/−^ *Bcr*-*Abl1* mouse model, BCR*::*ABL1-specific CD4+ memory T cells played a protective role, with T cell depletion drastically increasing leukemia outgrowth after dasatinib or cytotoxic chemotherapy (59). Similarly, protective T cell responses are generated by toll-like receptor-mediated immune modulation in the Eμ-ret model of hyperdiploid B-ALL (60). In addition, evidence that tolerance mechanisms affect the durability of T cell-mediated protection against Eμ-ret B-ALL outgrowth has been reported, suggesting that secondary, less immunogenic antigens might contribute to anti-leukemia T cell activity (61). However, the contribution of such antigens to ongoing immunosurveillance prior to disease presentation remains unknown.

Collectively, findings support a model of B-ALL progression in which an *in utero*-generated preleukemic cell population persists due to the undermining of effective immunosurveillance against the driver mutation by early-life tolerance mechanisms. The occurrence of secondary genomic lesions in preleukemic cells drives transformation but may also induce neoantigen-targeted immune responses against the emerging leukemia cells. According to this model, if T cell exhaustion is a pathway that enables immune escape and the emergence of overt leukemia, B-ALL blasts and patient T cells should be characterized by the expression of inhibitory IC molecules.

## 3 Clinical evidence of T cell exhaustion in pediatric B-ALL

The expression of exhaustion markers at various timepoints during pediatric B-ALL progression has been reported, summarized in **Table 1**. At diagnosis, there is an upregulation of IC receptors on patient T cells and their corresponding ligands on B-ALL blasts (68, 69, 71–73). Elevated expression of PD-1 and CTLA-4 have been observed on both αβ+ and γδ+ T cells in newly diagnosed ALL patients prior to chemotherapy (62). Notably, higher CTLA-4 levels on γδ+ T cells and CD86 expression on blasts has been linked to poor prognosis in high-risk B-ALL. Similarly, analysis of the bone marrow (BM) immune microenvironment in B-ALL showed increased expression of TIGIT, LAG3, and PD-1 on CD4+ and CD8+ T cells compared to healthy controls (63). In addition, several studies have reported upregulation of multiple immune checkpoint molecules at diagnosis across different compartments, including serum, peripheral blood mononuclear cells (PBMCs), and bone marrow mononuclear cells (BMMCs). These findings include elevated PD-L1 levels in the serum of children with ALL at diagnosis (67); upregulation of inhibitory molecules such as TIM-3, NR4A1, and BATF on CD8+ T cells in bone marrow aspirates from children with PAX5 mutation (64), and significantly higher TIM-3 mRNA expression in peripheral blood and BM of ALL patients (1.7- and 5-fold higher, respectively, compared to controls) (66). A bioinformatic analysis further suggested that increased expression of CD39, CTLA-4, TNFR2, TIGIT, and TIM-3 on Tregs and CD8+ T cells may contribute to disease progression (65).

**Table 1:** Overview of exhaustion markers on T cells from pediatric B-ALL patients at diagnosis, during treatment, and at relapse.

Research study	Disease stage (Dx or Relapse)	Cell source (PB, PBMC, BMMNC)	T cell subset	Markers of exhaustion	Clinical correlate
(62)	Newly diagnosed, prior to chemotherapy	PBMC	αβ and γδ T cells	PD-1 and CTLA-4 expression higher on αβ and γδ T cells. The expression of CTLA-4 on γδ T cells and B7-H2 ligand on blasts was higher in patients with high risk ALL.	Expression of CTLA-4 on γδ T cells and PD-L1 on ALL blasts are associated with poor prognosis in B-ALL.
(63)	Diagnosis	BM aspirates, BM MNCs	CD4+ and CD8+ T, Tregs	Increased expression of TIGIT, LAG3 and PD-1 on CD4 and CD8+T cells. T cells also had a higher proportion of Foxp3 expressing Treg cells.	
(64)	Not mentioned	Bone marrow	CD8+T cells	Tim-3, NR4A1 and BATF were upregulated in Pax5 haploinsufficient tumors.	
(65)	Not mentioned	mRNA from PBMCs	CD8+T cells, Tregs	Tim-3, TIGIT and CTLA-4 are overexpressed in B-ALL patients.	Increased Treg cells as well as CD8+T cells expressing CD39, CTLA-4, TNFR2, TIGIT and Tim-3 favor B-ALL progression.
(66)	Diagnosis	PB and BM	n/a	Relative mRNA expression of Tim-3 in PB and BMMNCs was 1.7 and 5 times higher in ALL patients compared to HD.	
(67)	Diagnosis	Blood serum	n/a	Elevated PD-1 in the serum of children with ALL compared to healthy volunteers	
(68)	Diagnosis	PBMC	CD8+T cells	PD-1 was upregulated on CD8+T cells in B-ALL patients	
(69)	End of induction chemotherapy	Matched PB and BM samples	CD8+T	At end of induction therapy, CD8+T cells from the bone marrow upregulated PD-1	
(70)	Newly diagnosed, prior to chemotherapy, 28 months after therapy and relapse.	PB	n/a	sCTLA-4 was upregulated in the serum of patients with B-ALL. Expression of serum sCTLA-4 was higher at relapse and in patients who died from disease.	High serum levels of sCTLA-4 and CD86 in B-ALL patients is a candidate parameter for poor prognosis.
(71)	Relapse	BM samples	CD4+T cells	Tim-3 is upregulated on CD4+T cells in patients with ALL	Expression of Tim-3 on CD4+T cells is a risk factor for disease relapse.
(72)	Diagnosis and Relapse	PBMC or Blasts	CD3+T cells	Expression of PD-L1 was higher in relapse patients compared to diagnosis on ALL blasts. PD-1 and Tim-3 was elevated on patients T cells compared to control.	

Elevated IC molecule expression continue to be detected after the initiation of B-ALL treatment. Evaluation of matched PB and BM samples from B-ALL patients post induction therapy indicated higher PD-1 expression on BM T cells, with PD-1 and LAG3 levels further upregulated on CD4+ and CD8+ T cells following ex vivo expansion (69). High circulating soluble CTLA-4 (sCTLA-4) levels have been detected in 70% of pediatric B-ALL patients with active disease (74), with elevated sCTLA-4 and CD86 levels associated with poor prognosis (70). In relapsed B-ALL following allogeneic hematopoietic stem cell transplantation (allo-HSCT), increased co-expression of PD-1 and TIM-3 on CD4+ and CD8+ T cells correlated with reduced proliferative capacity, cytokine production, and cytotoxic potential (73). Notably, the frequency of PD-1+TIM-3+ CD8+ T cells was lower in patients who achieved a complete remission. Lastly, while CD8+T cells are undoubtedly important mediators of anti-tumor immunity (75), recent studies suggest that exhausted CD4+ T cells may predict risk of relapse. In a study by Blaeschke et al., B-ALL was associated with a late-stage CD4+ phenotype, with high TIM-3 expression on BM CD4+ T cells correlating with a higher risk of relapse (71). Collectively, these findings suggest a functional relevance of IC expression on both CD4+ and CD8+ T cells during B-ALL development and relapse.

## 4 Pre-clinical support for functional T cell exhaustion in pediatric B-ALL

Preclinical studies using murine models of B-ALL have shed light on the role of ICs in disease progression. While exhausted CD8+ T cells are characterized by distinct functional, epigenetic and transcriptional features, many of these characteristics remain poorly defined for exhausted CD4+T cells. Recent work using primary patient samples and a murine model of Ph+ B-ALL have shown that phenotypic exhaustion predominantly occurs within a unique subset of CD4+ T cells (76). This subset, defined by its transcriptomic profile, displays hybrid functionality, exhibiting both cytotoxic and helper functions. In a syngeneic murine model of TCF3::PBX1 leukemia, an upregulation of PD-1, TIM-3, and LAG3 on CD4+ and CD8+ T cells was observed in the presence of leukemia (77). The resulting leukemia-induced T cell dysfunction was independent of TCR signaling and led to the generation of suboptimal autologous CAR-T cells, which were less effective in clearing leukemia blasts compared to CAR-T cells generated from naïve mice.

Further research has indicated that inhibiting myeloid–epithelial–reproductive tyrosine kinase (MERTK), a gene linked to the induction of an antiapoptotic gene expression signature in B-ALL cells, decreased PD-1 expression on both CD4+ and CD8+ T cells, leading to enhanced T cell activation and anti-ALL immune activity (78). Similarly, IL-12-mediated leukemia clearance in a syngeneic murine model of B-ALL was dependent on T cell activity. T cells from mice that failed to achieve leukemia clearance exhibited expression of exhaustion-associated genes, including LAG3 and TIGIT (79). Lastly, in a syngeneic model of Eμ−ret B-ALL, mice that failed to control non−immunogenic wild−type ALL blasts exhibited an upregulation of PD−1 and CTLA−4 on both CD4^+^ and CD8^+^ splenic T cells, whereas mice receiving B-ALL cells that express GFP/luciferase (which act as a model antigens) did not (80). This elevated checkpoint expression in non−responders was accompanied by higher CD80 on conventional dendritic cells and increased PD−L1 on plasmacytoid DCs. In contrast, T cells from leukemia−responsive mice downregulated these inhibitory receptors, allowing effective DC maturation, IL−12 production, and IFN−γ release by naïve T cells against otherwise non−immunogenic leukemia antigens. These results suggest that PD−1 and CTLA−4 inhibit epitope spreading and support combined checkpoint blockade strategies to broaden anti−ALL immunity.

In summary, increasing evidence from both clinical and preclinical studies indicates that B-ALL progression is accompanied by impaired T cell function, characterized by the overexpression of multiple IC molecules. This finding has significant implications for the application of immune therapies to children with B-ALL.

## 5 Immune checkpoint blockade in B-ALL

The role for immune checkpoint pathways in cancer progression, and rationale for ICB therapy, was first identified when antibodies targeting CTLA-4 demonstrated efficacy in reducing melanoma tumor size in mice (81). This early discovery led to the development of ipilimumab, an anti-CTLA-4 monoclonal antibody (mAb) that became the first therapy to improve survival in patients with metastatic melanoma (82). PD-1 then emerged as another critical immune checkpoint. Anti-PD-1/PD-L1 therapies, such as pembrolizumab and nivolumab, showed promising efficacy in controlling tumor progression, leading to their approval in 2014 for metastatic melanoma (83, 84). In general, ICB therapy with blocking mAbs has shown most success in solid tumors with high mutation loads, where it can achieve durable clinical responses (85). However, clinical efficacy is largely confined to a subset of patients (86). In the years since their approval, hundreds of clinical trials have explored the impact of ICB mAbs across diverse cancers, with varying degrees of success. In hematologic malignancies like B-ALL, the application of immune checkpoint inhibitors remains under investigation (87–89).

Given the low TMB and minimal neoantigen-specific T cell generation in pediatric B-ALL, ICB alone was predicted to be insufficient to achieve meaningful therapeutic activity. However, the recent findings described above have challenged this notion, prompting a re-examination of anti-ALL T cell activity. Preclinical B-ALL models show early evidence that ICB, alone or combined, can induce remissions. For instance, CTLA-4 blockade in Eμ-ret mice, which are likely tolerized to antigens derived from the leukemia-driving transgene, enhanced immune control and extended survival by 50% (61). In a BCR::ABL+ ALL mouse model, PD-L1 blockade led to clonal expansion of leukemia-specific CD4+T cells with a helper/cytotoxic phenotype, while reducing exhaustion marker expression (76). Additionally, combining PD-L1 mAb with nilotinib, a tyrosine kinase inhibitor (TKI), significantly improved survival of BCR::ABL+ leukemia-bearing mice. Studies with dasatinib, another TKI, in combination with anti-PD-1 eliminated BCR::ABL+ ALL cells, prolonged survival, and induced anti-leukemic immune memory upon rechallenge in syngeneic mice (90). These intriguing findings suggest that the administration of ICB during standard therapy is worthy of thorough preclinical investigation. One key question is whether targeting established or emerging exhaustion pathways (during immune reconstitution following induction chemotherapy) can enhance immune-mediated clearance of residual disease (91). Notably, a Phase 2 study of pembrolizumab for treating minimal residual disease (MRD) in adults with B-ALL found limited clinical benefit from anti-PD-1 therapy in this setting (92). However, given the distinct differences in immune reconstitution and treatment responses between adults and children, investigating this approach in pediatric B-ALL remains warranted. Finally, although B-ALL is characterized by low TMB, levels vary across different B-ALL subtypes. For instance, KMT2A-rearranged (KMT2A-r) ALL typically exhibits a low TMB, whereas iAMP21 ALL tends to have a comparatively higher TMB (93–95). Current data are insufficient to establish a clear association between TMB variations across B-ALL subtypes and their responsiveness to ICB therapy.

## 6 Integrating immune checkpoint blockade with approved immunotherapies

With the current shortage of empirical evidence supporting single-agent ICB use during pediatric B-ALL treatment, ongoing efforts are focused on exploring ICB in combination with other immunotherapies. Redirected T cell therapies, such as CAR-T and BiTE, facilitate cytotoxicity by directing autologous T cells toward leukemia cell surface antigens (96). Both CARs and BiTEs operate independently of the TCR and MHC molecules and rely on single-chain variable fragment (scFv) to recognize tumor-associated antigens (97). However, similar to peptide-specific T cells, continuous exposure of redirected T cells to tumor antigen can lead to T cell exhaustion (98), posing a significant challenge for both therapies. Emerging evidence supports that integration of ICB into these therapy protocols may achieve superior outcomes (99, 100).

### 6.1 ICB with CD3/CD19 BiTE

Blinatumomab, a CD3/CD19 BiTE, has notably improved outcomes for pediatric patients with relapsed or refractory (R/R) B-ALL and is now a frontline treatment option (101, 102). As of 2025, it remains the only immunotherapy approved for pediatric B-ALL patients who are minimal residual disease (MRD) positive, showing promise in low burden early-stage disease in combination with standard chemotherapy (103). Despite this, patient responses to blinatumomab can vary considerably. Some patients show little to no response to the treatment (104, 105), while others experience a loss of response after multiple cycles (104). Blinatumomab has been shown to induce an upregulation of inhibitory receptors on T cells and their corresponding ligands on B-ALL blasts, such as PD-1 and PD-L1, respectively. A comparison of IC expression at diagnosis and relapse showed higher PD-L1 expression on blasts obtained at relapse or from patients refractory to the anti-CD19 BiTE blinatumomab. Additionally, relapsed patients who failed to respond to blinatumomab exhibited increased expression of PD-1 and TIM-3 on T cells, alongside elevated PD-L1 on B-ALL blasts (71, 72). Recent data from a study of 11 pediatric patients treated with a continuous 28-day infusion of blinatumomab revealed progressive acquisition of T-cell exhaustion features (106). T cells exhibited phenotypic and transcriptomic upregulation of inhibitory receptors including PD-1, TIM-3, and TIGIT, a shift toward CD8^+^ T effector memory cells re-expressing CD45RA (TEMRA) subsets, and reduced cytotoxic and proliferative capacity. In a patient-derived xenograft (PDX) model of B-ALL using umbilical cord blood-reconstituted immunodeficient mice, treatment with either blinatumomab or pembrolizumab alone led to partial disease protection (107). Notably, the combination of both treatments resulted in a lower incidence of MRD and improved leukemia-free survival. Lastly, treatment of a single refractory ALL patient with a combination of blinatumomab and anti-PD-1 antibody induced anti-leukemic responses, reducing the disease burden from 45% to 1% (72).

### 6.2 ICB with CAR-T

CD19-directed CAR-T therapy has achieved over 70% complete remission rates in pediatric R/R B-ALL patients (108–111). However, 30–50% of these children experience relapse within the first year (112–114). Relapse in B−ALL patients following CAR−T therapy is most often driven by loss of leukemia-associated antigen (e.g., CD19 or CD22) or limited CAR−T cell persistence and proliferative capacity due to T cell exhaustion (115). In a syngeneic B−ALL mouse model, DeGolier et al. demonstrated that CD8^+^ CAR−T cells inherit epigenetic and transcriptional programs from their prior TCR antigen exposure, which dictate their exhaustion susceptibility (116). Although memory−derived CAR−T cells mount superior initial effector responses, they rapidly develop exhaustion phenotypes under low−antigen or low−dose conditions, which are marked by an increased expression of PD-1, TIM-3, Tox and CD39. In contrast, naive−derived CAR−T cells sustain proliferation and resist dysfunction. Complementing these findings, Zebley et al. analyzed CD8^+^ CD19-CAR T cells from pediatric B-ALL patients and found that ongoing antigen stimulation drives exhaustion-associated DNA methylation reprogramming (117). This includes demethylation at genes such as CX3CR1, BATF, and TOX, alongside repression of memory-associated genes like TCF7 and LEF1. This collectively promotes a progenitor-exhausted phenotype which limits CAR-T cell persistence. In a related study, it was shown that transiently interrupting CAR signaling—using either a drug-regulatable system or dasatinib—can restore functionality in exhausted CAR-T cells through epigenetic remodeling (118). This brief period of rest reprograms CAR-T cells toward a memory-like state, enhancing their cytokine production, proliferative potential, and antitumor efficacy.

As the limited long-term efficacy of CAR-T therapy has been linked to CAR-T cell exhaustion, ICB has been explored as a strategy to enhance persistence (119). In a small cohort study involving 14 pediatric B-ALL patients, the addition of anti-PD-1 mAbs to CD19 CAR-T cell therapy enhanced CAR-T cell persistence. Remarkably, three out of six patients treated with the PD-1 inhibitor at the time of early B cell recovery re-established B cell aplasia, signifying restoration of CAR-T cell activity (120). Other potential strategies for ICB integration with CAR-T therapy include engineering CAR-T cells to secrete soluble PD-1-blocking scFv. This approach has shown anti-leukemic efficacy comparable to combination therapy with CAR-T cells and anti-PD-1 antibodies (121), and has demonstrated improved eradication of CD19+PD-L1+ leukemia cells (122). Another approach involves the development of TIM-3-CD28 fusion proteins, which convert inhibitory TIM-3 signaling into an activating, immunostimulatory signal (123). However, the complex outcomes of ICB require further investigation, as increased CAR-T cell activation can reduce cell survival and exacerbate exhaustion by upregulating TIGIT (124).

A growing body of research emphasizes the significance of patient-derived T cell quality on CAR-T cell performance. Evaluating both the apheresis starting material and the post-infusion CAR-T product is crucial for identifying correlates of CAR-T cell persistence (125). Notably, increased expression of exhaustion markers, such as PD-1 and LAG-3, on CD8+ T cells within the apheresis material has been associated with reduced CAR-T cell efficacy (126). Conversely, higher levels of these markers on CD4+ CAR-T cells during peak expansion in recipients - triggered by cognate antigen-mediated activation - have been associated with prolonged event-free survival (127). These observations highlight that timing of IC assessments is crucial, as elevated expression at different times are associated with very different outcomes. A recent study examined the apheresis materials from pediatric and young adult patients with R/R B-ALL undergoing CD22 CAR-T cell therapy and found that T cells from non-responders had a more differentiated phenotype and overexpressed exhaustion-associated genes (128). These differences in the apheresis material could predict response to therapy, with the exhausted phenotype being a key predictor of poor outcomes. The study indicates that early identification of exhaustion markers in apheresis material could guide targeted manufacturing adjustments to optimize CAR-T cell efficacy.

### 6.3 Challenges to integration

Despite the promise of ICB, several translational and clinical barriers must be overcome if it is to become an established treatment in the pediatric setting. First, ICB-associated immune-related adverse events (irAEs), such as colitis, endocrinopathies, and hepatitis, represent a significant risk in children, whose immune and endocrine systems are still developing (129–131). The long-term effects of checkpoint inhibition on immune and organ development in children remain unknown. Second, the optimal timing and patient selection for ICB remain open questions. Introducing checkpoint inhibitors during immune reconstitution, such as post-chemotherapy or allo-HSCT, may either enhance anti-leukemic responses or disrupt essential tolerance pathways. Third, empirical evidence supporting the clinical use of ICB for pediatric B-ALL is limited. Most existing data are derived from adult trials or preclinical models, and few clinical trials that include pediatric B-ALL have been initiated (**Table 2**). These challenges highlight the importance of careful patient stratification, pediatric-specific trial design, and long-term monitoring if the field is to move toward clinical translation of ICB in B-ALL. These goals are made even more challenging to achieve by the diverstiy of clinical trials available for this patient population.

**Table 2:** Pediatric trials of ICB in B-ALL.

NCT (Trial)	ICB agent(s)	Combo agent(s)	Phase	Age (yrs)	Status	Objective/Eligibility
NCT05310591	Nivolumab (anti–PD-1)	CD19 CAR-T tisagenlecleucel (Kymriah^®^)	1	1-25	Recruiting	First relapse, to determine safety and efficacy of nivolumab with CD19 CAR-T
NCT04546399	Nivolumab (anti–PD-1)	Blinatumomab	II	≥ 1 to <31	Suspended – FDA partial clinical hold	First relapse, to compare event free survival post re-induction between blinatumomab vs blinatumomab/nivolumab
NCT03605589	Pembrolizumab (anti-PD-1	Blinatumomab	I/II	1-40	Withdrawn (lack of enrollment)	First relapse, to determine safety and feasibility of combining pembrolizumab with blinatumomab to treat relapsed B-ALL.

## 7 Future considerations

Over the past decade, it has become clear that T cell-based immunotherapies can overcome the hurdles of low immunogenicity and tolerance to achieve significant therapeutic activity in children with B-ALL. However, T cell exhaustion has emerged as a characteristic feature of B-ALL progression. This finding identifies ICB therapy as an important consideration for improved treatment of children with progressive disease. Several strategies to integrate ICB into current treatment regimens merit further investigation, including during immune reconstitution following chemotherapy and in combination with redirected T cell therapies. Considerable work will be needed to establish ICB as a central component of B-ALL therapy, but the continued application of both preclinical models and clinical studies to unveil underlying biology, identify the variables determining outcome, and optimize protocols could quickly set new immunotherapeutic standards that ultimately improving long-term outcomes for pediatric patients with B-ALL.
